# Environmental Factors for the Advancement of Teachers’ Self-Efficacy in Professional Development

**DOI:** 10.3390/jintelligence11020028

**Published:** 2023-01-31

**Authors:** Mehmet Hilmi Saglam, Talha Goktenturk, Ibrahim Demir, Emre Yazıcı

**Affiliations:** 1 Department of Educational and Counselling Psychology, and Special Education (ECPS), The University of British Columbia, Vancouver, BC V6T 1Z4, Canada; 2 Faculty of Education, Yıldız Technical University, Istanbul 34220, Turkey; 3 Faculty of Science, Yıldız Technical University, Istanbul 34220, Turkey

**Keywords:** teacher professional development, ecologically intelligent school, self-efficacy, structural equation modeling

## Abstract

There is a shifting paradigm in gifted education from person-based approaches (i.e., identifying giftedness) to process-based approaches (i.e., transacting giftedness). This new framework is centered on enriching educational opportunities that will make the process meaningful (i.e., gifted) to everyone in a setting. However, little is known about how this renewed perspective can be applied in teacher professional development. In line with the socio-ecological models, our study aims to identify the best appropriate model to describe teacher self-efficacy (i.e., the dependent variable in the study) as professional development from an ecological perspective and to propose an ecologically intelligent school (EIS) for the advancement of self-efficacy. Structural equation modeling (SEM) was performed to create a model using TALIS 2018 dataset. Afterward, indices of goodness-of-fit criteria were examined for each model. The results indicate that there is a complex ecological background, in that various factors affect the dependent variable. Model 3 was determined as the most suitable model that can be proposed as an ecologically intelligent school (EIS) for the advancement of self-efficacy. The factors within the three layers of the socio-ecological model—communication with teachers, communication with students, school climate, and feeling valued by the national level—altogether created an appropriate model explaining teacher professional development, regarding self-efficacy.

## 1. Introduction and Shifting Paradigm in Giftedness

The purpose of education must be focusing on and developing the talents and potentials of each stakeholder in the school, instead of merely focusing on the students (Daly-Smith et al. 2020). When contemplating any implementation related to school improvement and effectiveness, teachers should be given priority as the second-largest group in educational settings after students because teachers are the primary agents of change in schools (MacGilchrist et al. 2004). Therefore, the new paradigm in giftedness may enable improving teacher effectiveness by making teacher’s professional development more intelligent (i.e., gifted). This renewed perspective refers to a process of enriched educational opportunities that suit the students’ learning potential and needs (Lo et al. 2022), which may also offer a new way for individuals—including teachers—to meet their own needs (Peters et al. 2020). Considering complicated and real-world difficulties, the concept of giftedness has been moving from smart people to smart contexts (Plucker et al. 2021). Therefore, rather than concentrating on individuals, attention should shift to processes and situations (Lo et al. 2019; Peters et al. 2020). According to sociocultural theories, knowledge can be found in individuals, tools, and contexts, as well as in the human brain (Stetsenko and Arievitch 2004). With a sociocultural lens, educators ought to consider whether context is designed to allow teachers to develop their skills (Peters et al. 2020), since context is a matter for the quality and efficiency of education (Rose and Fischer 2011). In line with this theory, socio-ecological models (Bronfenbrenner 1979; McLeroy et al. 1988) can provide a framework for a better understand the ecological background of teachers’ professional development and make the process more gifted. This novel idea centers on a process of enriched educational opportunities for individuals (Lo et al. 2022). One way to enrich educational opportunities is to focus on teachers’ implicit theories of intelligence (Davidson and Kemp 2011) and teachers’ self-efficacy (Komarraju and Nadler 2013; Macakova and Wood 2022).

### 1.1. Implicit Theories of Intelligence & Self-Efficacy

The emphasis of Dweck’s idea of implicit theories of intelligence is on how a person’s implicit theories (i.e., mindsets) construct a framework for motivation and cognition that influences learning engagement and success (Bandura 2001). Growth and fixed mindsets are two different ways that people see intelligence, according to Dweck and her colleagues (Dweck 2000; Dweck et al. 1995). While entity theorists, who have a fixed mindset, think that intelligence is unalterable and stable, incremental theorists believe that intelligence is adjustable and improvable with effort (Dweck and Bempechat 2017; Hong et al. 1995). Incremental theorists consider that mastery can be attained through learning. For instance, they commonly use performance results as feedback to assess their dedication to the task at hand and their learning style (Liu 2021). On the other hand, entity theorists usually regard their level of intelligence as fixed, in light of performance criticism. When given negative criticism for their performance, people frequently generalize about their incapacity, quit too soon, and eventually weaken (Blackwell et al. 2007; Dweck and Bempechat 2017). Entity theorists contend that academic success indicates intelligence, while academic failure indicates lack of intelligence. Therefore, encouraging teachers to adopt a growth mindset is one of the most essential strategies for enhancing professional development opportunities and enhancing the efficiency of education. Improving self-efficacy is one strategy for accomplishing this (MacGilchrist et al. 2004). Studies have demonstrated that self-efficacy and growth mindsets (i.e., implicit theories) are highly correlated (Komarraju and Nadler 2013; Macakova and Wood 2022; Molway and Mutton 2020; Spicer 2017). Dweck (2013) asserted that beliefs about intelligence have an impact on people’s feelings of self-efficacy and their goals, self-regulation, and academic success (Komarraju and Nadler 2013). In comparison to other structures, self-efficacy is, especially, a multidimensional construct that conceptualizes people as being purposeful, proactive, self-evaluative, and self-regulatory (Bandura 1989). Self-efficacy is a motivational orientation that supports encouraging perseverance in the face of challenges, increases long-term planning, and promotes self-regulation and self-correcting behavior (Bandura 2001). In other words, it refers to a person’s beliefs of his or her own capacity to perform and accomplish goals (Bandura 1997; Macakova and Wood 2022). It is a subjective judgement on one’s ability, similar to mindsets in implicit theories (Bandura 1997). Self-efficacy is shaped by environmental influences through learning and using a variety of cognitive processes (Macakova and Wood 2022), which affect people’s beliefs about their own skills. Regarding teacher self-efficacy, it corresponds to teachers’ beliefs of their capacity to organize, plan, and carry out a task, in order to accomplish educational objectives (Klassen and Tze 2014; Skaalvik and Skaalvik 2007). The most important school variable that determines educational success is teacher quality (Hattie 2008). As a result, teachers’ beliefs about their ability and their professional knowledge can have a noteworthy positive impact on the atmosphere of school settings, educational goals, better learning, and teaching results (Chen et al. 2020). According to MacGilchrist et al. (2004), enhancing teachers’ self-efficacy beliefs can contribute to improving successful collaborative professional development in a school’s process. Therefore, the ecological background of teachers’ self-efficacy should be taken into consideration to better understand the structure of teacher professional development in school settings. Thus, better enriched environment can be built that match teachers’ needs, regarding a high level of self-efficacy, and therefore, can make the process more gifted (i.e., ecologically intelligent school with intelligent professional development).

### 1.2. Ecologically Intelligent School for the Advancement of Self-Efficacy

Self-efficacy is a complex and multifaceted structure that is influenced to varying degrees by a variety of factors. According to socio-ecological models (Bronfenbrenner 1979; McLeroy et al. 1988), any knowledge, attitudes, behavior, self-concept, or skills are influenced by both personal and social environmental factors in changing. For example, the ecological background of self-efficacy should be taken into account to evaluate and understand the structure of self-efficacy. The socio-ecological model by McLeroy et al. (1988) suggested that the behavior is determined and affected by the following levels or layers: (1) intrapersonal (individual) factors; (2) interpersonal processes; (3) institutional factors; (4) community factors; and (5) public policy. According to Gregson et al. (2001), the most specific level of influence is at the individual level (intrapersonal), which includes expressed behavioural choices, as well as cognitive and psychological factors, such as personality traits and beliefs. Regarding the interpersonal level, it refers to social networks consisting of family, friends, work groups, etc. Individuals’ attitudes and actions are influenced by a dynamic social environment in which they live with others (Pocock et al. 2012). For the institutional level, this includes organizational behaviours in public, private, and nonprofit sectors (Gregson et al. 2001) and how organizational characteristics can be used to support behavioral changes (McLeroy et al. 1988). The community level focuses on social networks, norms, and standards among individuals, groups, partnerships, and organizations (Voydanoff 2014). Finally, public policy, which is the social-ecological model’s highest level of influence, encompasses policy, social structure, and systems that regulate or support organizational or individual behavior (Gregson et al. 2001).

**Figure 1:**
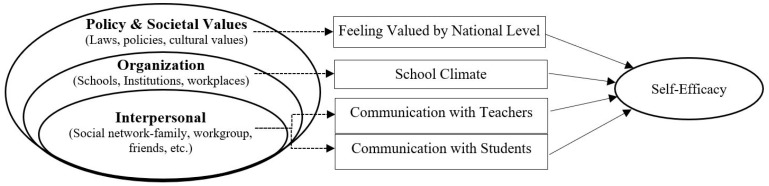
Conceptual Framework of Ecologically Intelligent School (EIS) for the Advancement of Self-Efficacy. Adapted from McLeroy et al. (1988) and Hu et al. (2021).

Considering the socio-ecological models and shifting paradigm in intelligent education, we propose a new model that may explain the structure of self-efficacy through socio-ecological perspective. By using these two unique perspectives, providing enrichment processes for the development of teachers’ self-efficacy (called “intelligent” appropriate to the literature above) and using socioecological perspective (ecologically) in school settings (school) generate the concept of the ecologically intelligent school (EIS). EIS is a framework that uses socio-ecological perspectives to enrich the school environment for the professionalism of teachers by addressing the issue of person–environment interactions. This fresh framework can serve as a roadmap for identifying the factors that could be enriched by opportunities, as well as the areas where the efficiency of teacher self-efficacy development should be increased. We named this model an ecologically intelligent school (EIS) for the advancement of self-efficacy. Therefore, this study provides which ecological factors related to teacher self-efficacy should be considered to make the process meaningful (gifted) for the development of teaching efficacy. In this study, we adopt the socio-ecological model by McLeroy et al. (1988) and reframe the model as three layers, as appropriate to the TALIS dataset (Hu et al. 2021; OECD 2019), in the following (shown in Figure 1): interpersonal, organization, and finally, policy and societal values.

Within the interpersonal layer of the EIS, the active communication environment is one of the essential components of teachers’ professional lives (Gordon 2010). Making meaningful connections with stakeholders is achievable when a teacher’s atmosphere is open to communication. Excellent school environments also depend on creating effective communication processes between students and teachers in the classrooms (Dhillon and Kaur 2021). Enhancing a teacher’s occupational environment, we must also concentrate on self-efficacy perceptions about their communication skills in the entire educational process (Kuru 2018; Kurudayıoğlu et al. 2021). Likewise, communication is a possible element for promoting self-efficacy (Green 2003). Therefore, we can see in-school communications as complex structure’s reflections that have great potential to contribute to an ecologically intelligent school. So, we evaluated this factor as a significant part of EIS, in line with the first layer.

School climate, which is impacted by the social and physical features of school settings (Slee and Skrzypiec 2016), is one concept in the organization layer of EIS. This term refers to quality level and character feature of a school life (Coulombe et al. 2020). The research demonstrated that the self-efficacy of teachers is positively influenced by a supportive environment at school (Daly-Smith et al. 2020; Meristo and Eisenschmidt 2014). Finally, the last layer within EIS is the policy and societal values. With an emphasis on high-need areas, the policy and societal values offer top-notch ongoing professional growth for teachers. Similar to the other components in the other layers above, teachers feeling valued at the national level has an immense influence on teachers’ self-efficacy perceptions (Price and McCallum 2015; Spruyt et al. 2021).

Considering all of the facts mentioned above, the model of an ecologically intelligent school (EIS) can help to understand behaviors, individual, and environmental determinants. Hence, we examined teachers’ self-efficacy to address the issue of person–environment interactions by employing an ecological perspective. This approach may contribute to enhancing the school process, so that teachers may be exposed by enriched (i.e., gifted) processes in school settings, which is identified above as EIS. By utilizing person–environment interactions, EIS may address and improve the issue of intelligent professional development. Therefore, this study specifically aims to find the best appropriate model to explain teacher self-efficacy from an ecological perspective and to propose a new model, called an ecologically intelligent school, for the advancement of self-efficacy. Our hypotheses are the following:

- Considering the conceptual framework (Figure 1), two factors together (i.e., *communication with teachers* (CT) and *with students* (CS)) in the interpersonal layer have a significant effect on teachers’ self-efficacy (Model 1).
- Three factors together (i.e., CT, CS, and *school climate* (SC)) have a significant effect on teacher self-efficacy (Model 2).
- Four factors together (i.e., CT, CS, SC, and *feeling valued by national level* (FV)) have a significant effect on teacher self-efficacy (Model 3—The Model of EIS).

The main research question for this study is: Which hypothesis mentioned above represents the most appropriate model (i.e., Model 1, Model 2, or Model 3) to explain the self-efficacy of teachers?

## 2. Materials and Methods

In this study, we used a quantitative approach with structural equation modeling (SEM) to accomplish the research’s aim. Accordingly, we explained the processes of analysis, the data source, samples, and variables.

### 2.1. Sample and Sampling Procedure of TALIS

The TALIS dataset, specifically upper secondary education (ISCED level 3), was used in this study (OECD 2019). In total, 38,081 participants from 11 countries took the survey. However, the country does not always specifically indicate an entire country. This term can also be the match of an OECD partner economy, an education system, a region, or a similar sub-national entity (Ainley and Carstens 2018). Descriptive statistics of the sample group are presented in Table 1 in alphabetical order.

TALIS data were created by a two-stage stratified cluster sampling. First stage includes the schools relevant to ISCED level. The second stage involves randomly sampled teachers (OECD 2019).

### 2.2. Factor Structures of Independent and Dependent Variables

In order to build a comprehensive model that characterizes the ecologically intelligent school (EIS) for the development of self-efficacy, three models were examined consecutively within the framework of the socio-ecological model (Kilanowski 2017). In the final model (Model 3), three layers (1. interpersonal; 2. organization; 3. public policy) helped to build the best suitable model for EIS (i.e., independent variables of the study). On the other hand, dependent variable of the research is self-efficacy. In parallel, the independent and dependent variables of the analysis were presented, respectively.

There are four factors in the final model. First, the *communication with teachers* (CT) factor has 4 items (TT3G32A, TT3G32B, TT3G32C, TT3G32D) and includes the communication process of teachers within colleagues. Additionally, the internal consistency is highly acceptable (α = .93). Second, the factor of *communication with students* (CS) has 5 items (TT3G49A, TT3G49B, TT3G49C, TT3G49D, TT3G49E) and explains communication process between teachers and students. Likewise, the internal consistency has a highly acceptable value (α = .85). Third, *school climate* (SC) has 8 items (TT3G48A, TT3G48B, TT3G48C, TT3G48D, TT3G48E, TT3G48F, TT3G48G, TT3G48H) and content to describe *school climate* (SC) (i.e., culture, management, encouragement). This factor’s internal consistency is .91, which is also an acceptable value. Final factor is the *feeling valued at the national level* (FV), which has 4 items (TT3G53H, TT3G54C, TT3G54D, TT3G54E). The internal consistency has a highly acceptable value (α = .84). No reverse coding was performed in the entire factors for independent variables because all items have a positive meaning.

Eigenvalues of items were provided in Appendix A. In SPSS data, answers were presented with a 4-point Likert scale, i.e., 1 = *strongly disagree*, 2 = *disagree*, 3 = *agree*, and 4 = *strongly agree* with all factors’ items. As a part of the validation, the correlation coefficients were also given among the factors and total structure that was obtained according to the final model (Model 3). Table 2 provides the correlation values among EIS for the development of self-efficacy and its factors, as well as their descriptive statistics.

As appropriate to our hypotheses, all factors given above are significantly correlated. Accordingly, these findings can be evaluated as an important evidence of validity (Odom and Morrow 2006) of the ecologically intelligent schools for the development of self-efficacy, which consists of 4 factors: *communication with teachers* (CT), *communication with students* (CS), *school climate* (SC), *perception of feeling valued at national level* (FV).

In dependent analysis, the explanatory factor analysis (EFA) provided two factorial structures for self-efficacy perceptions of teachers: *cognitive activation of students* (CAS) and *classroom management* (CM). First factor (CAS) has 8 items: TT3G34A, TT3G34B, TT3G34C, TT3G34G, TT3G34J, TT3G34K, TT3G34L, TT3G34M. The internal consistency value of the factor was found highly acceptable (α = .87). The second factor (CM) has 3 items: TT3G34D, TT3G34H, TT3G34I. This factor’s internal consistency value is also vastly acceptable (α = .84). Finally, the total internal consistency value of self-efficacy perceptions is highly acceptable (α = .89). In SPSS data, answers were presented with a 4-point Likert scale, i.e., 1 = *not at all*, *2* = *to some extent*, *3* = *quite a bit*, and *4* = *a lot* in all factors’ items. Table 3 represents the Pearson correlation results of self-efficacy and its factors, as well as their descriptive statistics.

In here, all factors of dependent variable (i.e., *self-efficacy of cognitive activation* (CAS) and *self-efficacy of classroom management* (CM)) are also significantly correlated. Therefore, it can be assessed as an evidence of total self-efficacy’s validity (Odom and Morrow 2006). In line with these factorial structures, the structural equation modeling (SEM) was made to discover the ecologically intelligent school’s effect on self-efficacy.

### 2.3. Data Analysis

There are a few important considerations that should be made before the major analysis procedure. First, the missing values are an all-pervasive issue in data analysis (Demir 2020; Field 2009). Second, there are two main analytical requirements for estimation in TALIS’ user guide: 1. sampling weights. 2. consideration of multi-stage cluster sample design (OECD 2019).

After the careful examination of CTGINTT3 file (i.e., upper secondary school data file), the missing value analysis was performed for each item of possible examination units. Selected units’ missing data percentage were: (1) between 2.3% and 2.4% in *communication with teachers* (CT); (2) between 2.6% and 2.8% in *communication with students* (CS); (3) between 2.7% and 3.1% in *school climate* (SC); (4) between 3.0% and 3.1% in *perception of feeling valued at national level* (FV); (5) between 2.5% and 2.8 in *self-efficacy* (SE).

Reaching reliable results and minimizing bias requires carefully handling missing data (Langkamp et al. 2010). There are several ways of dealing with this problem, such as deletion (Garson 2015) or imputation (Soley-Bori 2013). However, deletion or imputation also brings its own problems. First, the imputation method does not include the participants’ real answer. Second, the deletion method can do more harm to the sample size power of the study than the missing data (Davet and Savla 2010). Therefore, the dataset was accepted, as it received and included the analysis process.

In this study, we needed sampling weights to justify the inference both of our models parameters (Pfeffermann 1993) and fitting models to complex survey data (Pfeffermann 1996). TALIS provides the necessary sampling weights in the dataset. Therefore, there is no need to calculate another value separately. For the teacher-level analyses, a coded variable as TCHWGT that can be found on TTG-type files was selected (OECD 2019). This variable was used in our analysis procedure for SEM.

After completing the main analytical requirements, the first step was to discover the validity of possible factors that can affect self-efficacy based on the socio-ecological model. The levels of EIS were used as a guide to identify components in each model: 1. interpersonal. 2. organization. 3. public policy. Therefore, explanatory factor analysis (EFA) was performed. Maximum likelihood method was used with promax rotation technique because of their suitability to large datasets (Costello and Osborne 2005; de Winter and Dodou 2012; Dien 2010). Accordingly, Cronbach’s alpha values and Pearson correlation values were calculated. We used IBM SPSS 22 for the data preparation, explanatory factor analysis (EFA), Cronbach’s alpha calculation, and Pearson correlation coefficients.

The final structure of the independent and dependent variables was then validated using confirmatory factor analysis (CFA) on AMOS 22. The next stage involved determining whether multilevel analysis was required for this study. There are 11 countries in the upper secondary school level of TALIS. The initial group is formed by the individuals, and the subgroups are made up of 11 countries. To discover whether the multilevel was warranted, the intra-class correlation (ICC) was calculated based on 0.05 criterion (Geldhof et al. 2014) for dependent variable of research (i.e., self-efficacy of teachers). Based on the results of ICC, it was found that there is no need to perform multi-level structural equation modeling (MLSEM). Finally, 3 SEM models were tested, in order to find best model that can explain self-efficacy of teachers.

In order to check the goodness-of-fit criteria of the models (i.e., Model 1, Model 2, and Model 3), CMIN/df (Chi square/degree of freedom); RMSEA (root mean square error of approximation); CFI (comparative fit index); NFI (normed fit index), and TLI (Tucker–Lewis index) were examined. CMIN/df value is acceptable when these statistics are lower than 5 (Hooper et al. 2008). Chi-square statistics is considered sensitive to the large sample size (Barrett 2007). RMSEA value is also acceptable if below .08 (Browne and Cudeck 1992). CFI, NFI, and TLI was evaluated acceptable if above 0.90 (Shek and Yu 2014).

## 3. Results

Structural equation modelling (SEM) was used to examine the impact of two latent variables, namely *communication with teachers* (CT) and *communication with students* (CS), on self-efficacy perception in Model 1. Chi-square/degree of freedom (CMIN/df) is 176.45. The below 5 criteria are not met by this outcome (Hooper et al. 2008), but we should keep in mind that these statistics are sensitive to the sample size (Barrett 2007). However, the CFI (.97), TLI (.95), NFI (.97), and RMSEA (.07) indexes demonstrate a superior fit, in terms of model fit. These results are coherent with the theoretical framework. Both *communication with teachers* (CT) (Green 2003; Kuru 2018; Kurudayıoğlu et al. 2021) and *communication with students* (CS) (Dhillon and Kaur 2021) are potential elements to boost teachers’ self-efficacy.

*School climate* (SC) was added as a third latent variable to Model 2. The Chi-square/degree of freedom is 243.275. This finding must also be considered with the sensitivity about sample size (Barrett 2007). Other indexes—CFI (.92), NFI (.92), and RMSEA (.08)—indicate acceptable fit, in terms of the model fit. On the other hand, TLI (.89) value is not acceptable. This outcome led to the decision to modify the model by including more latent variables. As a result, the fourth latent variable, *feeling valued by national level* (FV), was added in the third SEM model.

**Figure 2:**
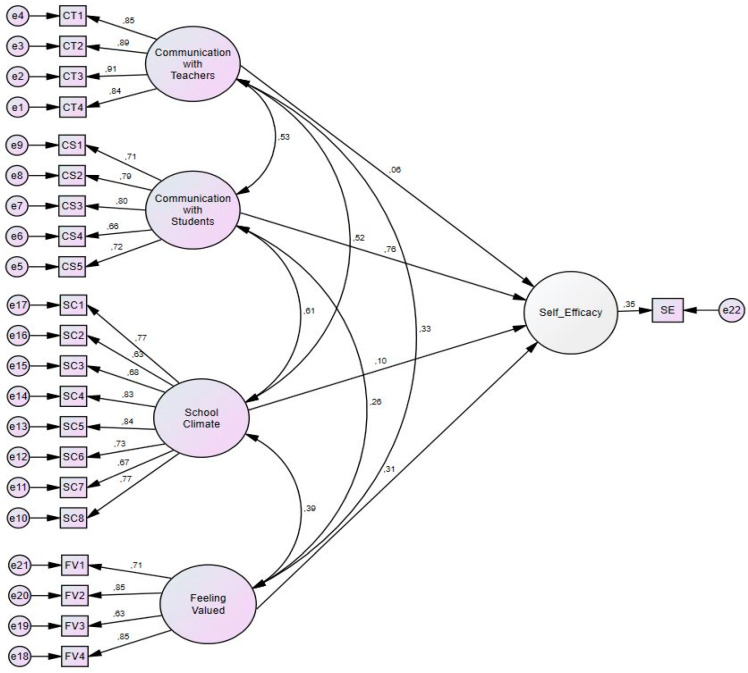
Path Diagram of Model 3 with Standardized Estimation.

Although Model 3’s Chi-square/degree of freedom value of 189.046 does not seem to be a good fit, we may safely disregard this result, due to the sensitivity issue mentioned above (Barrett 2007; Vandenberg 2006). On the other hand, the goodness-of-fit indexes—CFI (.92), TLI (.90), NFI (.92), and RMSEA (.07)—show a good fit. Therefore, the model was accepted as valid. Path analysis of the Model 3 was given in Figure 2. Parameter estimates and standardized factor loadings were presented, as well.

Finally, our SEM analysis revealed four latent variables—*communication with teachers* (CT), *communication with students* (CS), *school climate* (SC), and *feeling valued at the national level* (FV)—that explain the self-efficacy of teachers.

As a framework of EIS, this model proves that each has a significant effect on self-efficacy. *Communication with students* (SC) has explained noticeably higher *self-efficacy* (SE) than other independent variables (COV(CS-SE) = .76). Therefore, students can be evaluated as the most crucial point of teachers’ self-efficacy perceptions. All independent variables have positive covariance in the interaction with self-efficacy, which means they all have the potential to make a teacher’s environment better, as a part of an ecologically intelligent school (EIS). Another important aspect of the results is that open communication with the stakeholders of the school, a positive school environment, and feeling valued at the national level demonstrate positive results together. Accordingly, we must remember that a school is a complex structure, as a result of mixed combination of environments and members (Trombly 2014). Finally, Model 3 gives a more comprehensive frame to the socio-ecological background of self-efficacy than the other two models.

Hypothesis 1 (Model 1) and Hypothesis 3 (Model 3) were confirmed, while hypothesis 2 (Model 2) was rejected in Table 4. Model 1 has the best fit indexes among the models. However, the theoretical background must always be taken into consideration (Fan et al. 2016). The most comprehensive model that explains self-efficacy in a broader structure is Model 3. Hence, the final model with four latent variables was chosen because it best explained the socio-ecological background of teachers’ self-efficacy, as appropriate to the framework of an ecologically intelligent school.

## 4. Discussion

An increasing number of studies is emerging to discover the socio-ecological background of self-efficacy, and they indicate a complex background (Lamarche et al. 2018; Viel-Ruma et al. 2010). Likewise, the main purpose of this study is to build a model that can comprehensively describe the socio-ecological background of self-efficacy via an ecologically intelligent school (EIS). Three hypotheses were tested by using structural equation modeling. Model 3 (Hypothesis 3) was selected as the best appropriate model that can explain the background of self-efficacy. Model 3 presented four factors that can contribute this investigation area: *communication with teachers* (CT), *communication with students* (CS), *school climate* (SC), and *feeling valued at national level* (FV). Each result will be discussed, respectively.

*Communication with teacher* (CT) has little positive effect on teacher’s self-efficacy (COV(CS-SE) = .06). However, *communication with students* (CS) has the greatest effect among other factors (COV(CS-SE) = .76). These results can be interpreted a reflection of active communication’s significance in school environment (Gordon 2010). The enhancement of communication skills are mostly related to the students (Dhillon and Kaur 2021), and we can interpret the difference of covariance values accordingly. Several studies have the necessary perspective regarding the evaluation of self-efficacy with structural and environmental factors (Fackler et al. 2021; Holzberger et al. 2013; Sun and Xia 2018; Vieluf et al. 2013). The unique aspect of this study is that it gathers them under a more general heading and explicitly looks at how EIS affects self-efficacy. As appropriate to the literature (Lin et al. 2002; Özder 2011; Swackhamer et al. 2009; Yada and Savolainen 2017), the results from the first layer support Hypothesis 1, in that communication with teachers (CS) and communication with students (CS) together have a significant impact on teacher self-efficacy. Therefore, these two factors should be taken into account, in order to make teachers’ professional development more enriched (i.e., gifted).

*School climate* (SC) is the only factor of the organization layer of EIS. Structural equation modelling (SEM) findings revealed that a key component of environmentally intelligent schools is the school climate, which is a significant explanatory variable of self-efficacy (Meristo and Eisenschmidt 2014). It also influences all stakeholders (i.e., students, teachers, principals, parents) and being affected by them constantly (Thapa et al. 2013). Further, OECD also brings forward the same argument (Schleicher 2018), and several studies investigate it with a socio-ecological perspective (Long et al. 2021; Marraccini et al. 2020). However, this study evaluates the school climate as a part of the ecologically intelligent school’s framework, instead of not being a variable examined alone. As far as the authors’ knowledge, this is a new look for the school climate to describe it with EIS. As a result, it can support the goal of promoting self-efficacy as a component of a broad viewpoint.

Our study also revealed that teachers’ perception of *feeling valued at the national level* (FV) is another component of the EIS’s structure for the development of self-efficacy. Multiple studies report that one of the most beneficial point of teachers’ professional development is *feeling valued at the national level* (Asbury and Kim 2020; Price and McCallum 2015; Spruyt et al. 2021). Further, this variable is also benign for well-being (Farley and Chamberlain 2021). In all examined studies, feeling valued is considered within a limited frame. However, our findings provide an opportunity of a unique perspective to combine these elements with a complex layer into one school of understanding (i.e., ecologically intelligent school).

Here, it is important to note that *school climate* (SC) and *feeling valued at the national level* (FV) showed a better fit together. This is also another supportive finding that schools are not only exist with their own ecosystem, but they are also affected by a complex environment (Boeve-de Pauw and Van Petegem 2011; Johnson 2009; Ucci et al. 2015). Our results for school climate and feeling valued at the national level are also other indicators of a supportive environment’s positive influence (Meristo and Eisenschmidt 2014). In this kind of climate, stakeholders and teachers can contribute to the school process with roles such as principals in school management (Rizvi 2008; Sağlam and Alpaydın 2017).

The central argument of this study is to determine EIS’s effect on self-efficacy, which is highly correlated with the growth mindset (Komarraju and Nadler 2013; Macakova and Wood 2022; Molway and Mutton 2020; Spicer 2017). We also created a beginning point for the adoption of the enhancement of self-efficacy via an EIS. In the guidance of our results, the enriched educational opportunities (Lo et al. 2022) and considering the teachers’ own needs (Peters et al. 2020) helps to improve self-efficacy. In conclusion, the EIS has shown the potential to offer a new path regarding how a school can shape its own atmosphere in a smart context (Plucker et al. 2021). As an inevitable and powerful workforce, teachers consist of the foundation of a school environment and help their students to accomplish their goals (Sun and Xia 2018). Therefore, schools, administrators/principals, and policymakers can provide an ecologically intelligent school to the teachers by enriching these ecological factors and helping them to enhance their self-efficacy. Last, but not least, the framework of EIS can be viewed as a guideline for stakeholders on how to improve self-efficacy levels in teacher development.

## Figures and Tables

**Table 1:** Descriptive statistics of the sample group.

Country	Frequency [N—Number]	Percent [%]
Alberta (Canada)	1094	2.9
Brazil	2828	7.4
Chinese Taipei	2800	7.4
Crotia	2661	7
Denmark	1670	4.4
Portugal	3551	9.3
Slovenia	2200	5.8
Sweden	2933	7.7
Turkey	8342	21.9
United Arab Emirates	6118	16.1
Vietnam	3884	10.2
Total	38,081	100

**Table 2:** Correlation coefficients and descriptive statics of factors of EIS.

	CT	CS	SC	FV	EIS (Total)	M	SD	Range
**CT**	1					2.82	.46	1–4
**CS**	.73 **	1				2.94	.68	1–4
**SC**	.86 **	.48 **	1			2.86	.56	1–4
**FV**	.74 **	.49 **	.56 **	1		3.20	.49	1–4
**EIS (Total)**	.64 **	.31 **	.37 **	.25 **	1	2.13	.76	1–4

Abbreviations: communication with teachers (CT), communication with students (CS), school climate (SC), feeling valued at national level (FV), ecologically intelligent school (EIS). **. Correlation is significant at the *p* < 0.01.

**Table 3:** Correlation coefficients and descriptive statics of dependent variable.

	CAS	CM	SE	M	SD	Range
**CAS**	1			3.28	.51	1–4
**CM**	.61 **	1		3.34	.59	1–4
**SE**	.97 **	.80 **	1	3.29	.48	1–4

Abbreviations: self-efficacy of cognitive activation (CAS), self-efficacy of classroom management (CM), total self-efficacy (SE). **. Correlation is significant at the *p* < 0.01.

**Table 4:** Summary of goodness-of-fit statistics from three SEM models in the study.

Layer in the Socio-Ecological Model	SEM Model	χ^2^/df	CFI	TLI	NFI	RMSEA
Layer 1	Model 1	176.45	.97	.95	.97	.07
Layer 2	Model 2	243.275	.92	.89	.92	.08
Layer 3	Model 3	189.046	.92	.90	.92	.07
Criterion for goodness-of-fit			≥0.90	≥0.90	≥0.90	≤0.08

Note: χ^2^/df, Chi-square/degree of freedom; CFI, comparative fit index; TLI, Tucker and Lewis’s index of fit; NFI, normed fit index; RMSEA, root mean square error of approximation.

## Data Availability

TALIS data are open source. So, any researcher who wants to reach the dataset can find it at https://www.oecd.org/education/talis/talis-2018-data.htm.

## Appendix A

Explanatory factor analysis (EFA), Cronbach’s alpha coefficient, and confirmatory factor analysis (CFA) findings of dependent and independent variables can be reached by using the following link. The list of the selected TALIS items is also presented below. Further, the QR code is provided, just in case the link has an error. https://drive.google.com/drive/folders/11-9GrVKZHn3NIuY09eU9pwxJGjgtBIiX?usp=sharing.

## References

[B1-jintelligence-11-00028] Ainley John, Carstens Ralph (2018). Teaching and Learning International Survey (TALIS) 2018 Conceptual Framework.

[B2-jintelligence-11-00028] Asbury Kathryn, Kim Lisa E. (2020). ‘Lazy, Lazy Teachers’: Teachers’ Perceptions of How Their Profession Is Valued by Society, Policymakers, and the Media during COVID-19.

[B3-jintelligence-11-00028] Bandura Albert (1989). Human Agency in Social Cognitive Theory. American Psychologist.

[B4-jintelligence-11-00028] Bandura Albert (1997). Self Eflicacy. The Exercise of Control, New York: WH. Freeman & Co. Student Success.

[B5-jintelligence-11-00028] Bandura Albert (2001). Social Cognitive Theory: An Agentic Perspective. Annual Review of Psychology.

[B6-jintelligence-11-00028] Barrett Paul (2007). Structural Equation Modelling: Adjudging Model Fit. Personality and Individual Differences.

[B7-jintelligence-11-00028] Blackwell Lisa S., Trzesniewski Kali H., Dweck Carol Sorich (2007). Implicit Theories of Intelligence Predict Achievement Across an Adolescent Transition: A Longitudinal Study and an Intervention. Child Development.

[B8-jintelligence-11-00028] Boeve-de Pauw Jelle, Van Petegem Peter (2011). The Effect of Flemish Eco-schools on Student Environmental Knowledge, Attitudes, and Affect. International Journal of Science Education.

[B9-jintelligence-11-00028] Bronfenbrenner Urie (1979). The Ecology of Human Development: Experiments by Nature and Design.

[B10-jintelligence-11-00028] Browne Michael W., Cudeck Robert (1992). Alternative Ways of Assessing Model Fit. Sociological Methods & Research.

[B11-jintelligence-11-00028] Chen Robin Jung-Cheng, Lin Hsin-Chih, Hsueh Yi-Lung, Hsieh Chuan-Chung (2020). Which Is More Influential on Teaching Practice, Classroom Management Efficacy or Instruction Efficacy? Evidence from TALIS 2018. Asia Pacific Education Review.

[B12-jintelligence-11-00028] Costello Anna B., Osborne Jason (2005). Best Practices in Exploratory Factor Analysis: Four Recommendations for Getting the Most from Your Analysis. Practical Assessment, Research, and Evaluation.

[B13-jintelligence-11-00028] Coulombe Simon, Hardy Kendra, Goldfarb Rachel (2020). Promoting Wellbeing through Positive Education: A Critical Review and Proposed Social Ecological Approach. Theory and Research in Education.

[B14-jintelligence-11-00028] Daly-Smith Andy, Quarmby Thomas, Archbold Victoria S. J., Corrigan Nicola, Wilson Dan, Resaland Geir K., Bartholomew John B., Singh Amika, Tjomsland Hege E., Sherar Lauren B. (2020). Using a Multi-Stakeholder Experience-Based Design Process to Co-Develop the Creating Active Schools Framework. International Journal of Behavioral Nutrition and Physical Activity.

[B15-jintelligence-11-00028] Davet Adam, Savla Jyoti (2010). Statistical Power Analysis with Missing Data: A Structural Equation Modeling Approach.

[B16-jintelligence-11-00028] Davidson Janet E., Kemp Iris A., Sternberg Robert J., Kaufman Scott Barry (2011). Contemporary Models of Intelligence. The Cambridge Handbook of Intelligence.

[B86-jintelligence-11-00028] de Winter J. C. F., Dodou D. (2012). Factor Recovery by Principal Axis Factoring and Maximum Likelihood Factor Analysis as a Function of Factor Pattern and Sample Size. Journal of Applied Statistics.

[B17-jintelligence-11-00028] Demir İbrahim (2020). SPSS Ile Istatistik Rehberi.

[B18-jintelligence-11-00028] Dhillon Navdeep, Kaur Gurvinder (2021). Self-Assessment of Teachers’ Communication Style and Its Impact on Their Communication Effectiveness: A Study of Indian Higher Educational Institutions. SAGE Open.

[B19-jintelligence-11-00028] Dien Joseph (2010). Evaluating Two-Step PCA of ERP Data with Geomin, Infomax, Oblimin, Promax, and Varimax Rotations. Psychophysiology.

[B20-jintelligence-11-00028] Dweck Carol S. (2000). Self-Theories.

[B21-jintelligence-11-00028] Dweck Carol S. (2013). Self-Theories: Their Role in Motivation, Personality, and Development.

[B22-jintelligence-11-00028] Dweck Carol S, Bempechat Janine (2017). Children’s Theories of Intelligence: Consequences for Learning. Learning and Motivation in the Classroom.

[B23-jintelligence-11-00028] Dweck Carol S., Chiu Chi-yue, Hong Ying-yi (1995). Implicit Theories and Their Role in Judgments and Reactions: A Word From Two Perspectives. Psychological Inquiry.

[B24-jintelligence-11-00028] Fackler Sina, Malmberg Lars-Erik, Sammons Pamela (2021). An International Perspective on Teacher Self-Efficacy: Personal, Structural and Environmental Factors. Teaching and Teacher Education.

[B25-jintelligence-11-00028] Fan Yi, Chen Jiquan, Shirkey Gabriela, John Ranjeet, Wu Susie R., Park Hogeun, Shao Changliang (2016). Applications of Structural Equation Modeling (SEM) in Ecological Studies: An Updated Review. Ecological Processes.

[B26-jintelligence-11-00028] Farley Amy N., Chamberlain Leah M. (2021). The Teachers Are Not Alright: A Call for Research and Policy on Teacher Stress and Well-Being. The New Educator.

[B27-jintelligence-11-00028] Field Andy (2009). Discovering Statistics Using SPSS.

[B28-jintelligence-11-00028] Garson G. David (2015). Missing Values Analysis and Data Imputation.

[B29-jintelligence-11-00028] Geldhof G. John, Preacher Kristopher J., Zyphur Michael J. (2014). Reliability Estimation in a Multilevel Confirmatory Factor Analysis Framework. Psychological Methods.

[B30-jintelligence-11-00028] Gordon Thomas, Karakale Sermin (2010). Etkili Öğretmenlik Eğitimi.

[B31-jintelligence-11-00028] Green Denise M. (2003). Self-Efficacy: A Communication Model for the Development of Self-Efficacy in the Classroom. Journal of Teaching in Social Work.

[B32-jintelligence-11-00028] Gregson Jennifer, Foerster Susan B, Orr Robin, Jones Larry, Benedict Jamie, Clarke Bobbi, Hersey James, Lewis Jan, Zotz Karen (2001). System, Environmental, and Policy Changes: Using the Social-Ecological Model as a Framework for Evaluating Nutrition Education and Social Marketing Programs with Low-Income Audiences. Journal of Nutrition Education.

[B33-jintelligence-11-00028] Hattie John (2008). Visible Learning: A Synthesis of over 800 Meta-Analyses Relating to Achievement.

[B34-jintelligence-11-00028] Holzberger Doris, Philipp Anja, Kunter Mareike (2013). How Teachers’ Self-Efficacy Is Related to Instructional Quality: A Longitudinal Analysis. Journal of Educational Psychology.

[B35-jintelligence-11-00028] Hong Ying-yi, Chiu Chi-yue, Dweck Carol S. (1995). Implicit Theories of Intelligence. Efficacy, Agency, and Self-Esteem.

[B36-jintelligence-11-00028] Hooper Daire, Coughlan Joseph, Mollen Michael R. (2008). Structural Equation Modelling: Guidelines for Determining Model Fit. Electronic Journal on Business Research Methods.

[B37-jintelligence-11-00028] Hu Donglin, Zhou Shi, Crowley-McHattan Zachary J., Liu Zhiyun (2021). Factors That Influence Participation in Physical Activity in School-Aged Children and Adolescents: A Systematic Review from the Social Ecological Model Perspective. International Journal of Environmental Research and Public Health.

[B38-jintelligence-11-00028] Johnson Sarah Lindstrom (2009). Improving the School Environment to Reduce School Violence: A Review of the Literature. Journal of School Health.

[B39-jintelligence-11-00028] Kilanowski Jill F. (2017). Breadth of the Socio-Ecological Model. Journal of Agromedicine.

[B40-jintelligence-11-00028] Klassen Robert M, Tze Virginia MC (2014). Teachers’ Self-Efficacy, Personality, and Teaching Effectiveness: A Meta-Analysis. Educational Research Review.

[B41-jintelligence-11-00028] Komarraju Meera, Nadler Dustin (2013). Self-Efficacy and Academic Achievement: Why Do Implicit Beliefs, Goals, and Effort Regulation Matter?. Learning and Individual Differences.

[B42-jintelligence-11-00028] Kuru Oğuzhan (2018). Analysis of Classroom Teaching Candidates’ Speaking Self-Efficacy in Terms of Different Variables. International Journal of Education and Literacy Studies.

[B43-jintelligence-11-00028] Kurudayıoğlu Mehmet, Yazıcı Emre, Göktentürk Talha (2021). Turkish Teacher Candidates’ Self-Efficacies to Use Listening Strategies Scale: A Validity and Reliability Study. SAGE Open 11.

[B44-jintelligence-11-00028] Lamarche Larkin, Tejpal Ambika, Mangin Dee (2018). Self-Efficacy for Medication Management: A Systematic Review of Instruments. Patient Preference and Adherence.

[B45-jintelligence-11-00028] Langkamp Diane L., Lehman Amy, Lemeshow Stanley (2010). Techniques for Handling Missing Data in Secondary Analyses of Large Surveys. Academic Pediatrics.

[B46-jintelligence-11-00028] Lin Huey-Ling, Gorrell Jeffrey, Taylor Janet (2002). Influence of Culture and Education on U. S. and Taiwan Preservice Teachers’ Efficacy Beliefs. The Journal of Educational Research.

[B47-jintelligence-11-00028] Liu Woon Chia (2021). Implicit Theories of Intelligence and Achievement Goals: A Look at Students’ Intrinsic Motivation and Achievement in Mathematics. Frontiers in Psychology.

[B48-jintelligence-11-00028] Lo C. Owen, Lin-Yang Rachel C., Chrostowski Megan (2022). Giftedness as a Framework of Inclusive Education. Gifted Education International.

[B49-jintelligence-11-00028] Lo C. Owen, Porath Marion, Yu Hsiao-Ping, Chen Chen-Ming, Tsai Kuei-Fang, Wu I-Chen (2019). Giftedness in the Making: A Transactional Perspective. Gifted Child Quarterly.

[B50-jintelligence-11-00028] Long Emily, Zucca Claudia, Sweeting Helen (2021). School Climate, Peer Relationships, and Adolescent Mental Health: A Social Ecological Perspective. Youth & Society.

[B51-jintelligence-11-00028] Macakova Viviana, Wood Clare (2022). The Relationship between Academic Achievement, Self-Efficacy, Implicit Theories and Basic Psychological Needs Satisfaction among University Students. Studies in Higher Education.

[B52-jintelligence-11-00028] MacGilchrist Barbara, Reed Jane, Myers Kate (2004). The Intelligent School.

[B53-jintelligence-11-00028] Marraccini Marisa E., Fang Yumeng, Levine Sharon P., Chin Andrew J., Pittleman Cari (2020). Measuring Student Perceptions of School Climate: A Systematic Review and Ecological Content Analysis. School Mental Health.

[B54-jintelligence-11-00028] McLeroy Kenneth R, Bibeau Daniel, Steckler Allan, Glanz Karen (1988). An Ecological Perspective on Health Promotion Programs. Health Education Quarterly.

[B55-jintelligence-11-00028] Meristo Merilyn, Eisenschmidt Eve (2014). Novice Teachers’ Perceptions of School Climate and Self-Efficacy. International Journal of Educational Research.

[B56-jintelligence-11-00028] Molway Laura, Mutton Trevor (2020). Changing Mindsets in the Modern Foreign Languages Classroom: An Intervention Combining Intelligence Theories and Reading Strategies. The Language Learning Journal.

[B57-jintelligence-11-00028] Odom Leslie R., Morrow James R. (2006). What’s This r? A Correlational Approach to Explaining Validity, Reliability and Objectivity Coefficients. Measurement in Physical Education and Exercise Science.

[B58-jintelligence-11-00028] OECD (2019). TALIS 2018 and TALIS Starting Strong 2018 User Guide.

[B59-jintelligence-11-00028] Özder Hasan (2011). Self-Efficacy Beliefs of Novice Teachers and Their Performance in the Classroom. Australian Journal of Teacher Education.

[B60-jintelligence-11-00028] Peters Scott J., Carter James, Plucker Jonathan A. (2020). Rethinking How We Identify ‘Gifted’ Students. Phi Delta Kappan.

[B61-jintelligence-11-00028] Pfeffermann Danny (1993). The Role of Sampling Weights When Modeling Survey Data. International Statistical Review.

[B62-jintelligence-11-00028] Pfeffermann Danny (1996). The Use of Sampling Weights for Survey Data Analysis. Statistical Methods in Medical Research.

[B63-jintelligence-11-00028] Plucker Jonathan A., McWilliams Jacob, Guo Jiajun, Sternberg Robert J., Ambrose Don (2021). Smart Contexts for 21st Century Talent Development. Conceptions of Giftedness and Talent.

[B64-jintelligence-11-00028] Pocock Barbara, Williams Philippa, Skinner Natalie (2012). Conceptualizing Work, Family and Community: A Socio-Ecological Systems Model, Taking Account of Power, Time, Space and Life Stage: Conceptualizing Work, Family and Community. British Journal of Industrial Relations.

[B65-jintelligence-11-00028] Price Deborah, McCallum Faye (2015). Ecological Influences on Teachers’ Well-Being and ‘Fitness’. Asia-Pacific Journal of Teacher Education.

[B66-jintelligence-11-00028] Rizvi Meher (2008). The Role of School Principals in Enhancing Teacher Professionalism: Lessons from Pakistan. Educational Management Administration & Leadership.

[B67-jintelligence-11-00028] Rose L. Todd, Fischer Kurt W., Sternberg Robert J., Kaufman Scott Barry (2011). Intelligence in Childhood. The Cambridge Handbook of Intelligence.

[B68-jintelligence-11-00028] Sağlam Mehmet Hilmi, Alpaydın Yusuf (2017). The Relationship between School Administrators Personalities and Servant Leadership Behaviours. Journal of Education and Training Studies.

[B69-jintelligence-11-00028] Schleicher Andreas (2018). PISA 2018 Insights and Interpretations.

[B70-jintelligence-11-00028] Shek Daniel T. L., Yu Lu (2014). Confirmatory Factor Analysis Using AMOS: A Demonstration. International Journal on Disability and Human Development.

[B71-jintelligence-11-00028] Skaalvik Einar M, Skaalvik Sidsel (2007). Dimensions of Teacher Self-Efficacy and Relations with Strain Factors, Perceived Collective Teacher Efficacy, and Teacher Burnout. Journal of Educational Psychology.

[B72-jintelligence-11-00028] Slee Phillip T., Skrzypiec Grace (2016). Well-Being at School. Well-Being, Positive Peer Relations and Bullying in School Settings.

[B73-jintelligence-11-00028] Soley-Bori Marina (2013). Dealing with Missing Data: Key Assumptions and Methods for Applied Analysis.

[B74-jintelligence-11-00028] Spicer Margaret I. (2017). A Correlational Study of the Relationships between Implicit Theories of Intelligence, Perceived Self-Efficacy, Self-Regulated Learning, and Academic Achievement of Undergraduate Students at an HBCU. Doctoral Dissertation.

[B75-jintelligence-11-00028] Spruyt Bram, Droogenbroeck Filip Van, Borre Laura Van Den, Emery Laura, Keppens Gil, Siongers Jessy (2021). Teachers’ Perceived Societal Appreciation: PISA Outcomes Predict Whether Teachers Feel Valued in Society. International Journal of Educational Research.

[B76-jintelligence-11-00028] Stetsenko Anna, Arievitch Igor M. (2004). The Self in Cultural-Historical Activity Theory: Reclaiming the Unity of Social and Individual Dimensions of Human Development. Theory & Psychology.

[B77-jintelligence-11-00028] Sun Anna, Xia Jiangang (2018). Teacher-Perceived Distributed Leadership, Teacher Self-Efficacy and Job Satisfaction: A Multilevel SEM Approach Using the 2013 TALIS Data. International Journal of Educational Research.

[B78-jintelligence-11-00028] Swackhamer Lyn Ely, Koellner Karen, Basile Carole, Kimbrough Doris (2009). Increasing the Self-Efficacy of Inservice Teachers through Content Knowledge. Teacher Education Quarterly.

[B79-jintelligence-11-00028] Thapa Amrit, Cohen Jonathan, Guffey Shawn, Higgins-D’Alessandro Ann (2013). A Review of School Climate Research. Review of Educational Research.

[B80-jintelligence-11-00028] Trombly Christopher Edmund (2014). Schools and Complexity. Complicity: An International Journal of Complexity and Education.

[B81-jintelligence-11-00028] Ucci Marcella, Law Stephen, Andrews Richard, Fisher Abi, Smith Lee, Sawyer Alexia, Marmot Alexi (2015). Indoor School Environments, Physical Activity, Sitting Behaviour and Pedagogy: A Scoping Review. Building Research & Information.

[B82-jintelligence-11-00028] Vandenberg Robert J. (2006). Introduction: Statistical and Methodological Myths and Urban Legends: Where, Pray Tell, Did They Get This Idea?. Organizational Research Methods.

[B83-jintelligence-11-00028] Viel-Ruma Kim, Houchins David, Jolivette Kristine, Benson Gwen (2010). Efficacy Beliefs of Special Educators: The Relationships among Collective Efficacy, Teacher Self-Efficacy, and Job Satisfaction. Teacher Education and Special Education: The Journal of the Teacher Education Division of the Council for Exceptional Children.

[B84-jintelligence-11-00028] Vieluf Svenja, Kunter Mareike, van de Vijver Fons J.R. (2013). Teacher Self-Efficacy in Cross-National Perspective. Teaching and Teacher Education.

[B85-jintelligence-11-00028] Voydanoff Patricia (2014). Work, Family, and Community.

[B87-jintelligence-11-00028] Yada Akie, Savolainen Hannu (2017). Japanese In-Service Teachers’ Attitudes toward Inclusive Education and Self-Efficacy for Inclusive Practices. Teaching and Teacher Education.

